# Machine Learning for Fast, Quantum Mechanics-Based Approximation of Drug Lipophilicity

**DOI:** 10.1021/acsomega.2c05607

**Published:** 2023-01-04

**Authors:** Clemens Isert, Jimmy C. Kromann, Nikolaus Stiefl, Gisbert Schneider, Richard A. Lewis

**Affiliations:** † Department of Chemistry and Applied Biosciences, ETH Zurich, Vladimir-Prelog-Weg 4, 8093Zurich, Switzerland; ‡ Novartis Institutes for BioMedical Research, 4056Basel, Switzerland; § ETH Singapore SEC Ltd., 1 CREATE Way, #06-01 CREATE Tower138602, Singapore, Singapore

## Abstract

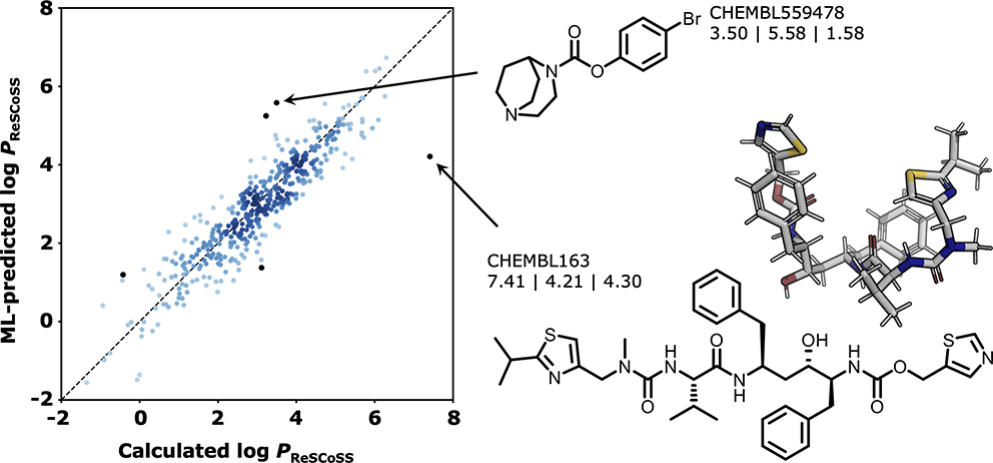

Lipophilicity, as measured by the partition coefficient between octanol and water (log *P*), is a key parameter in early drug discovery research. However, measuring log *P* experimentally is difficult for specific compounds and log *P* ranges. The resulting lack of reliable experimental data impedes development of accurate in silico models for such compounds. In certain discovery projects at Novartis focused on such compounds, a quantum mechanics (QM)-based tool for log *P* estimation has emerged as a valuable supplement to experimental measurements and as a preferred alternative to existing empirical models. However, this QM-based approach incurs a substantial computational cost, limiting its applicability to small series and prohibiting quick, interactive ideation. This work explores a set of machine learning models (Random Forest, Lasso, XGBoost, Chemprop, and Chemprop3D) to learn calculated log *P* values on both a public data set and an in-house data set to obtain a computationally affordable, QM-based estimation of drug lipophilicity. The message-passing neural network model Chemprop emerged as the best performing model with mean absolute errors of 0.44 and 0.34 log units for scaffold split test sets of the public and in-house data sets, respectively. Analysis of learning curves suggests that a further decrease in the test set error can be achieved by increasing the training set size. While models directly trained on experimental data perform better at approximating experimentally determined log *P* values than models trained on calculated values, we discuss the potential advantages of using calculated log *P* values going beyond the limits of experimental quantitation. We analyze the impact of the data set splitting strategy and gain insights into model failure modes. Potential use cases for the presented models include pre-screening of large compound collections and prioritization of compounds for full QM calculations.

## Introduction

Lipophilicity is of critical importance in early-stage drug discovery as it impacts a compound’s distribution between different body tissues and is related to several drug-relevant properties, such as membrane permeability,^1,2^ solubility,^2,3^ bioavailability,^4^ and metabolic stability.^5,6^ The octanol–water partition coefficient log *P* is a commonly used measure of lipophilicity, describing the distribution of a compound in the biphasic system (eq 1)
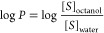
1where [*S*] is the concentration of the unionized (neutral) compound.^5^ While log *P* describes the distribution of the unionized compound, log *D* describes the distribution of both ionized (in different protonation states) and unionized species and thus depends on the pH value.^5,7^ Similar to the SAMPL6 log *P* challenge,^7^ this work focused on the prediction of log *P*. This isolates effects of predicting protonation states and p*K*_a_ values,^7^ and the quantum–mechanical method of choice (see the Methods section) has previously been tested for log *P* prediction.^8^

Numerous computational approaches for the prediction of log *P* have been developed.^9,10^ We provide a brief overview here and point toward more detailed descriptions elsewhere.^5,7,11^ Computational approaches to log *P* prediction can be grouped into (i) empirical and (ii) physics-based methods.^5,7^ Empirical methods (i) include contribution-type approaches (atom-^12^ or fragment-based^13,14^), QSAR approaches,^15^ and deep learning approaches^1,10,16−23^ trained on experimental data. Contribution-type approaches obtain a log *P* estimate by dividing molecules into either individual atoms or fragments and summing up their contributions, using correction terms in the latter case.^11^ QSAR and deep learning approaches fit model parameters to a set of training compounds with known experimental labels. Physics-based methods (ii) include, for example, semi-empirical,^24^ continuum–solvation,^9^ and molecular dynamics simulation^25^ approaches. While empirical approaches have shown competitive performance in recent blind log *P* prediction challenges (SAMPL6 and SAMPL7),^5,7^ the performance of such methods relies heavily on the scope of the training data. Specifically, they require accurate experimental measurements of molecules from the chemical space of interest.^26^

Experimental methods to obtain these log *P* measurements can be broadly categorized into two classes: direct or indirect methods. Direct methods, such as the shake-flask and slow-stir methods, obtain a log *P* measurement from the concentration ratio between water and octanol.^27^ Indirect methods obtain a log *P* measurement by measuring a correlated quantity such as capacity factors or solute retention volumes.^23,27,28^ The shake-flask and slow-stir methods are generally considered the gold standard for log *P* measurement.^26^ Experimental uncertainty estimates for log *P* measurements range from <0.2 to 0.4 log units and depend on the specific experimental setup.^23,29,30^

However, both direct and indirect methods struggle to measure log *P* for poorly soluble, very lipophilic (high log *P*), or very hydrophilic (low log *P*) compounds.^26^ This is well-illustrated by the relatively large fraction of compounds in the in-house data set with a measured log *P* annotated as “> 4.7” (Figure S1), highlighting the limits of the used experimental method (see the Methods section). Compounds in the extreme ranges of the log *P* scale are challenging to measure as either the limit of detection in the low-concentration phase may be approached or the detector in the high-concentration phase may be oversaturated.^23,26,31−33^ Cross-phase contamination in the layer sampling process may pose additional challenges.^33^ The use of a buffer solution to obtain a log *P* measurement of the neutral compound^34^ can further contribute to experimental uncertainty for such compounds. While successful measurements can be performed with complex experimental setups,^23,26,35,36^ reliable training data for highly lipophilic compounds is generally lacking.^26^ This may pose a particular challenge given the pharmaceutical industry’s increased interest in beyond-Rule-of-5 compounds in recent years and the substantial number of highly lipophilic compounds recently approved by the Food and Drug Administration (FDA).^2^ The lack of reliable, experimentally determined training data for compounds in this space renders the exploration of alternative means of obtaining training data for ML models attractive. In this study, we explore ReSCoSS^8^/COSMO-RS,^37−40^ a quantum mechanics (QM)-based method for log *P* estimation to generate synthetic training data for ML models.

For certain in-house drug discovery projects at Novartis, this log *P* estimation approach was found to be useful, particularly when existing empirical methods did not provide satisfactory results. The approach combines the “Relevant Solution Conformer Sampling and Selection” tool^8^ (ReSCoSS) with thermodynamic calculations using the “Conductor-like Screening Model for Real Solvents” (COSMO-RS).^37−40^ ReSCoSS is used to generate conformers and select relevant solution conformers from the resulting ensemble. Based on those conformers, COSMO-RS calculates log *P* from the free energy difference of the solute in water and octanol by considering the distribution of screening charge density (“sigma-profile”) around a molecule.^39^ For a range of approved drugs, COSMO-RS calculations based on ReSCoSS-generated conformers showed considerable performance improvement over a single-conformer approach.^8^ However, the relatively high computational cost associated with the ReSCoSS workflow (∼1 h/compound with semiempirical geometry optimization and four CPU cores) limits the tool’s use to smaller sets of compounds and prohibits quick, interactive use by medicinal chemists.

In this study, a range of different molecular representations and machine learning (ML) methods are analyzed to approximate ReSCoSS-calculated log *P* values at a substantially reduced computational cost. We focus on log *P* instead of log *D* to isolate the challenge of predicting compound lipophilicity from protonation states, similar to the approach taken in the SAMPL6 challenge.^7^ To assess the model accuracy, we compare the ML predictions both against ReSCoSS-calculated log *P* values and against experimental data. We aim to gain insights into the model failure modes by analyzing individual predictions and seek to determine the amount of training data required to build robust models by investigating learning curves.^41^

## Methods

### Data Sets

Two data sets were used: A public data set deposited by AstraZeneca^42^ in the ChEMBL database^43^ and a set of Novartis in-house compounds. As the public data set contains experimentally measured log *D* (pH = 7.4) values, MoKa^44−47^ (version 3.2.1, Molecular Discovery Ltd., enhanced with in-house data) was used to determine which molecules in the public data set would predominantly be present as neutral, non-zwitterionic species at pH = 7.4. The analysis was limited to these structures since log *P* = log *D* for unionized species.^7^ If multiple experimental measurements existed for the same compound, the arithmetic average of all values was used. Experimental log *P* values in the in-house data set were obtained using a previously described miniaturized shake-flask procedure.^8^ All compounds for which the measured log *P* value contained a qualifier (“<”/“>”) were excluded. The final public and in-house data sets used contained 2114 and 25,642 molecules, respectively. Both data sets contain appreciable fractions of beyond-Rule-of-5 compounds^48^ (∼7.4 and ∼20.1% for the public and in-house data sets, respectively). Figure S22 provides additional details on the number of Rule-of-5 violations. 29 compounds are present in both data sets, and the calculated log *P*_ReSCoSS_ values agree down to numerical precision, while experimental values generally show good consistency between both data sets (see Figure S23 for details).

### ReSCoSS Calculations

The ReSCoSS workflow was slightly changed with new approximations to increase computational efficiency as described in the following and depicted in Figure S19. The original workflow took ∼9 h/compound, which was cut down to ∼1 h/compound with the updated workflow. Furthermore, the workflow was automated to include tautomer enumeration.

To obtain an ReSCoSS calculated log *P* value per molecule, a two-step approach was used: first, different molecular configurations were evaluated for each compound by expanding the tautomer states and determining the lowest energy through a preliminary conformer evaluation (using UNICON^49^ version 2021 and GFN2-xTB^50−53^). As a design choice, this tautomer selection was performed in water, and performing it in octanol might result in a different tautomer subset for individual species. Low-energy tautomers were selected by including any tautomer within 7 kcal/mol of the overall lowest energy configuration. For each low-energy tautomer, protomers were generated using MoKa^44−47^ (version 3.2.1, Molecular Discovery Ltd., enhanced with in-house data).

For each molecule, charged or zwitterionic graphs were excluded (unless the charge separation was located in resonance-stabilized nitro groups, *N*-oxides, or azides). Undefined stereocenters were assigned using CORINA^54−56^ (version 4.4.0). If more than two stereocenters were unassigned in the input SMILES, the structure was discarded as being too unspecific. We note that since the experimental environment of a log *P* measurement (biphasic water/octanol system) is achiral, enantiomers can, in principle, not be distinguished and show the same log *P* value,^57^ while diastereomers would generally differ in observed log *P* values.

For conformer selection, we initially generated 1000 conformers using OpenEye OMEGA.^58,59^ These conformers were then filtered based on shape diversity as in the original ReSCoSS^8^ workflow, but using RDKit^60^ for calculating the solvent accessible surface area and dipole moment based on Gasteiger charges.^61^ To all unique conformers in shape space, a combination of outlier detection (IsolationForest) and clustering function (SKLearn DBSCAN) was applied.^62^ With the conformers filtered, they were optimized using GFN2-xTB^50−53^ in an implicit water solvent [using GFN2-xTB’s analytical linearized Poisson–Boltzmann (ALPB) model]. After optimization, the shape diversity is evaluated again as some of the conformers might have converged into the same local minima, using the same technique as before.

For each unique conformer, Turbomole^63−65^ (version 7.5.1) was used for single-point density functional theory (DFT) (BP86/def2-TZVPD) calculations,^66,67^ followed by COSMO-RS/COSMOtherm^37−40,68^ calculation for solvent effects. Based on previous experience, log *P*_ReSCoSS_ values > 8 were discarded as likely outliers. For a more detailed description of computational details regarding the DFT single-point calculations, please refer to the ReSCoSS paper.^8^

### Featurization and Models

We investigated three classes of models: 2D descriptor-based models (Random Forest,^69^ Lasso,^70^ and XGBoost^71^), 2D graph-based models (Chemprop^18,72^), and 3D graph-based models (Chemprop3D, for the public data set only). Random Forest, Lasso, and XGBoost models were trained using RDKit2DNormalized descriptors obtained from the DescriptaStorus^73^ package, concatenated with radius 2 Morgan Fingerprints^74^ (2,048 bit vector, useCounts = True) computed using RDKit.^60^

#### Chemprop

Chemprop is a directed message-passing neural network model that has shown strong performance on multiple tasks related to absorption, distribution, metabolism, excretion, and toxicity.^10,18^ As a graph-based deep learning architecture, Chemprop operates on a molecular graph consisting of nodes, which represent atoms, and edges, which represent chemical bonds. The network aggregates features based on the local chemical environment by iteratively propagating messages along the molecular graph using an edge-centric message passing algorithm. After message passing, atom hidden states are obtained by summing up the incoming edge hidden states, concatenating them with the respective atom features, and passing them through a feed-forward neural network layer. Finally, the readout phase aggregates the atom hidden states (using a mean operator in the case of the intensive quantity log *P*) and uses a multi-layer feed-forward neural network to output the final prediction. Chemprop models were trained using the package’s default featurization, which includes atom- (e.g., atomic number, degree, and formal charge) and bond- (e.g., bond type and ring membership) level features.^18,72^

#### Chemprop3D

Since the ReSCoSS pipeline produces 3D conformers, which are used for the COSMO-RS log *P* calculation, we investigated whether using this 3D information in our model predictions would result in an increased accuracy. 3D message-passing neural networks trained on large quantities of reference calculations^75,76^ have shown a strong performance for geometry-related tasks, particularly in predicting the QM properties such as energies or dipole moments.^77−80^ Here, we adapt the Chemprop architecture to include edges between all pairs of atoms within a specified cutoff distance from each other. This cutoff distance is a hyperparameter optimized during cross-validation (see the Supporting Information for details). To endow the model with a geometric understanding, the default Chemprop edge features are augmented with edge lengths for all edges in the molecular graph (bond lengths for bonded atoms; interatomic distances for nonbonded atoms). A similar 3D version of Chemprop has been used for the prediction of drug activity against SARS-CoV and SARS-CoV-2 3CL protease in the past.^81^ We chose the lowest GFN2-xTB^50−53^ computed energy in water solvent (ALPB implicit solvent) as the criterion for conformer choice and investigated the impact of using other metrics (GFN2-xTB energy in octanol; DFT computed energy in water) on model performance. In early experiments, exploding gradients^82^ were observed due to the increased number of edges in the graph when using not only edges based on covalent bonds but also edges based on interatomic distance. To alleviate this issue, the aggregation operation was modified so that atom hidden states *h*_v_ are obtained after message passing by using a mean rather than a sum operation over incoming edges. A batch size of 16 was used to ensure sufficient GPU memory for model training. Chemprop3D models were trained only for the public data set due to computational cost considerations and similar observed performance compared to the 2D Chemprop models (see the Results and Discussion section).

### Training Details and Hyperparameter Optimization

For each model, we report errors on a hold-out test set, containing 20% of the entire data set. Hyperparameters are optimized using a five-fold cross-validation scheme on the remaining 80%, choosing the set of hyperparameters that minimize the average mean absolute validation error over the different folds (see the Supporting Information for details).

Three ways of assigning molecules to the individual data set splits were investigated: a random split, a scaffold split, and a time split (for the in-house data set only). While being the easiest, the random split risks overestimating model performance since very similar compounds may end up in both the training and validation sets. A scaffold split groups molecules together on the basis of molecular scaffolds to decrease chemical similarity between the training and test sets, though compounds with similar decorations but different scaffolds may be placed in different splits. Analogous to the previous work,^1,18^ for the scaffold split, molecules were clustered by their Bemis–Murcko scaffolds^83^ and the resulting clusters were randomly assigned to the individual splits. A time split assigns molecules to training, validation, and test sets based on, for example, their registration date. Time split is considered the gold standard^84^ (in that it most closely mimics a real-world use case and most accurately assesses future model performance) but is not generally applicable to public data sets as time stamps are typically missing. A time split may not result in a strict split along chemical series as individual series may have been worked on for longer durations.^85^ For the time split, molecules were ordered chronologically based on their internal registration date, and splits were assigned so that molecules in the training set are registered before those in the validation set, and those in turn were registered before molecules in the test set.

By creating learning curves,^41^ we investigate the dependence of the test set error on training set size to understand whether additional data points are expected to increase the model accuracy. Since we train on computed values, models are not limited by experimental uncertainty and could, at least in theory, achieve a perfect predictive accuracy. For this analysis, we use increasingly larger training set sizes, with each larger set including the smaller ones. This enables us to isolate the effect of adding additional data points to the training set while removing potential impacts from how the training set was sampled. Hyperparameters are again optimized in a fivefold cross-validation scheme.

### Software

Python^86^ 3.8.13 along with its scientific software stack (NumPy^87^ 1.22.3, scikit-learn^62^ 1.0.2, Pandas^88^ 1.4.2) was used for data analysis. Figures were generated using Matplotlib^89^ 3.5.2, PyMOL^90^ 2.3.2, and ChemDraw. Models were trained using PyTorch^91^ 1.11.0. RDKit^60^ 2022.03.2 was used for molecular processing. XGBoost^71^ (version 1.5.2) was used for XGBoost models. Chemprop^18,72^ (version 1.5.1) was used for MPNN models. A wrapper for bridging cheminformatics (RDKit) and quantum chemistry was developed, which resulted in two Python packages—one for internal use including business rules and another with open-source availability.^92^ The code and data (public data set) used in this project are available at https://github.com/cisert/rescoss_logp_ml (resp. https://github.com/ETHmodlab/rescoss_logp_ml and https://doi.org/10.5281/zenodo.7500726).

## Results and Discussion

### Predictive Performance

**Figure 1:**
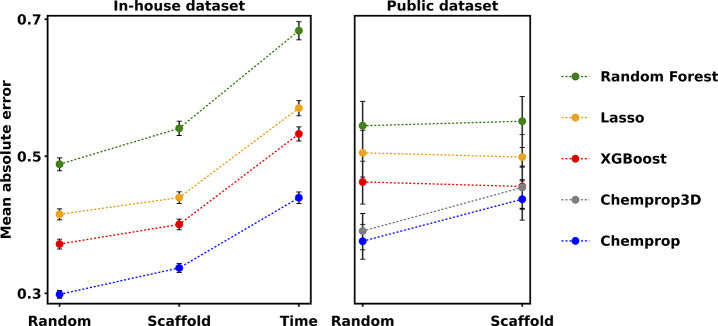
Test set errors for the in-house and public data sets with different data-splitting strategies. Error bars show 95% confidence intervals.^93^

Hold-out test set performance for both the public and in-house data sets is shown in Figure 1 (see Tables S2–S4 for more details). For both data sets and irrespective of the splitting strategy, the test set error decreased in the order Random Forest > Lasso > XGBoost > Chemprop, though the trends are more pronounced for the in-house data set, with Chemprop3D showing similar performance to Chemprop for the public data set. Test set errors (for the individual models and splitting procedures) were generally lower for the models trained and tested on the in-house data set than for those trained and tested on the public data set. This could be connected to two factors: training set size and chemical diversity. Regarding training set size, the in-house training set is larger than the public training set (ca. 20,500 and 1700 compounds, respectively). The dependence of the test set error on the training set size is further discussed in the section “Learning curves.” Regarding chemical diversity, compounds in the in-house data set show a higher mean Tanimoto similarity of a compound to its five nearest neighbors than compounds in the public data set (∼0.71 and ∼0.57, respectively, see Figure S21 for details). This higher similarity may contribute to lower test set errors for the in-house data set. The larger error bars (indicating 95% confidence intervals) in the public data set compared to the in-house data set are due to the differing test set sizes between both data sets.

For the in-house data set, models trained with a time split show a substantially higher test set error than models trained with a random or scaffold split. This illustrates the difficulty of extrapolating to new chemical series or subsets of chemical space that were only recently explored within a company. While slightly higher test set errors are observed for models trained with a scaffold split instead of a random split, the performance difference is generally quite small. This effect is likely caused by a high number of Bemis–Murcko fragments that occur only once in the data set (Table S1). Since, analogously to previous work,^1,18^ the individual scaffold clusters are randomly assigned to the training/validation/test set, the large number of single-member scaffold clusters will result in a behavior similar to that of a random split. For the public data set, the Random Forest, Lasso, and XGBoost see essentially no change in the test set error between a random split and a scaffold split. Meanwhile, the Chemprop and Chemprop3D models see increased test set errors for this scenario, potentially indicating that these graph-based models can exploit the higher train test similarity in the case of random splitting better than the other tested models.

**Figure 2:**
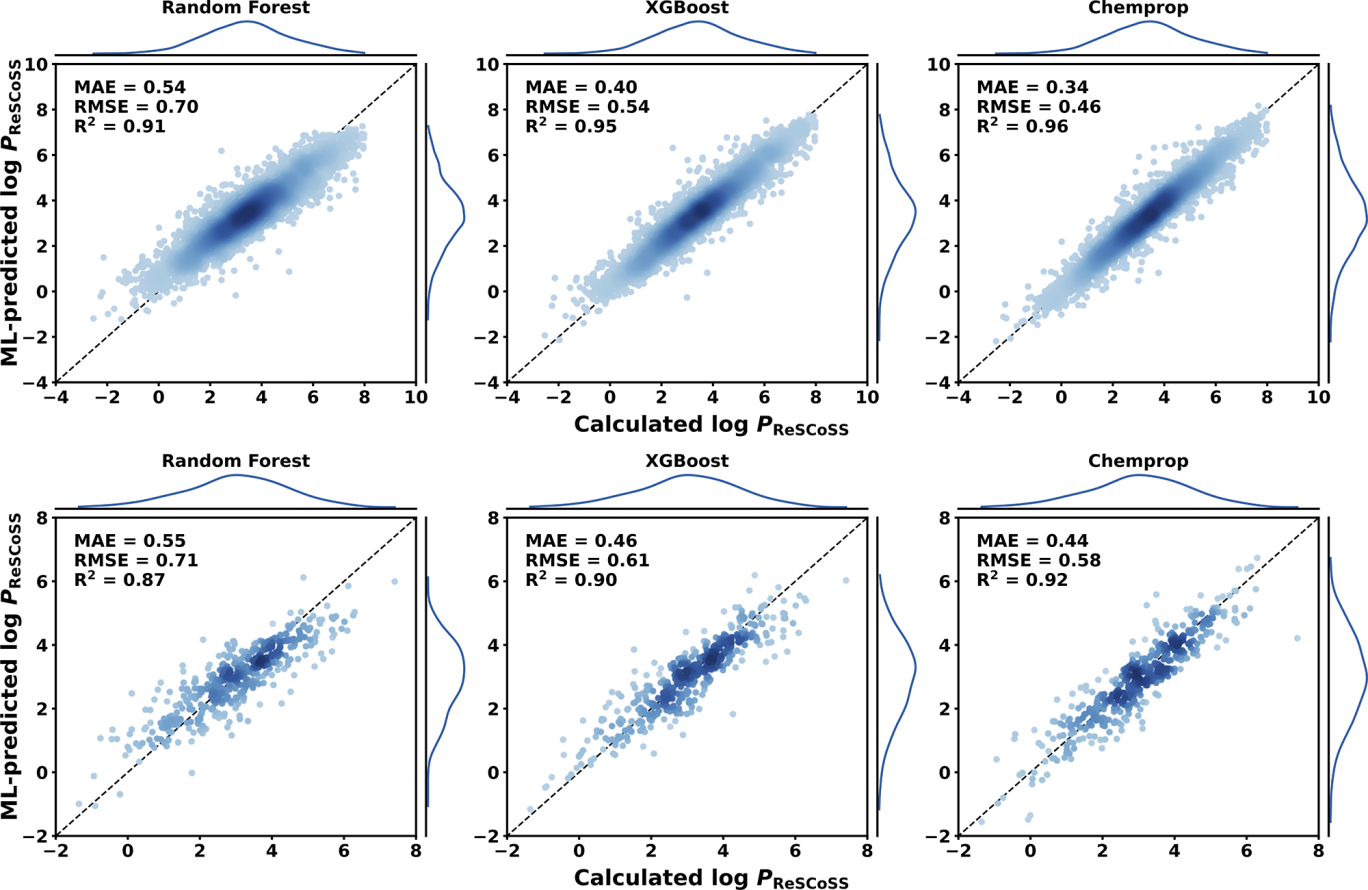
Predicted vs calculated plots for test set molecules. (Top): In-house data set. (Bottom): Public data set. Models trained using a scaffold-based split. Point density ranges from high (dark blue) to low (light blue). Kernel density estimates for predicted and calculated values are shown on the left and top of each panel, respectively.

Figure 2 shows predicted versus calculated plots for the Random Forest, XGBoost, and Chemprop models for both data sets using a scaffold split (see Figure S5–S9 for remaining models and splits). The model predictions lie around the unity line, and no clear trend to over- or underestimate calculated log *P*_ReSCoSS_ values is observed in any particular region of log *P* values. For the in-house data set, the lower test set error of the Chemprop models than that of the Random Forest and XGBoost models is clearly visible in the narrower distribution of points around the unity line.

**Figure 3:**
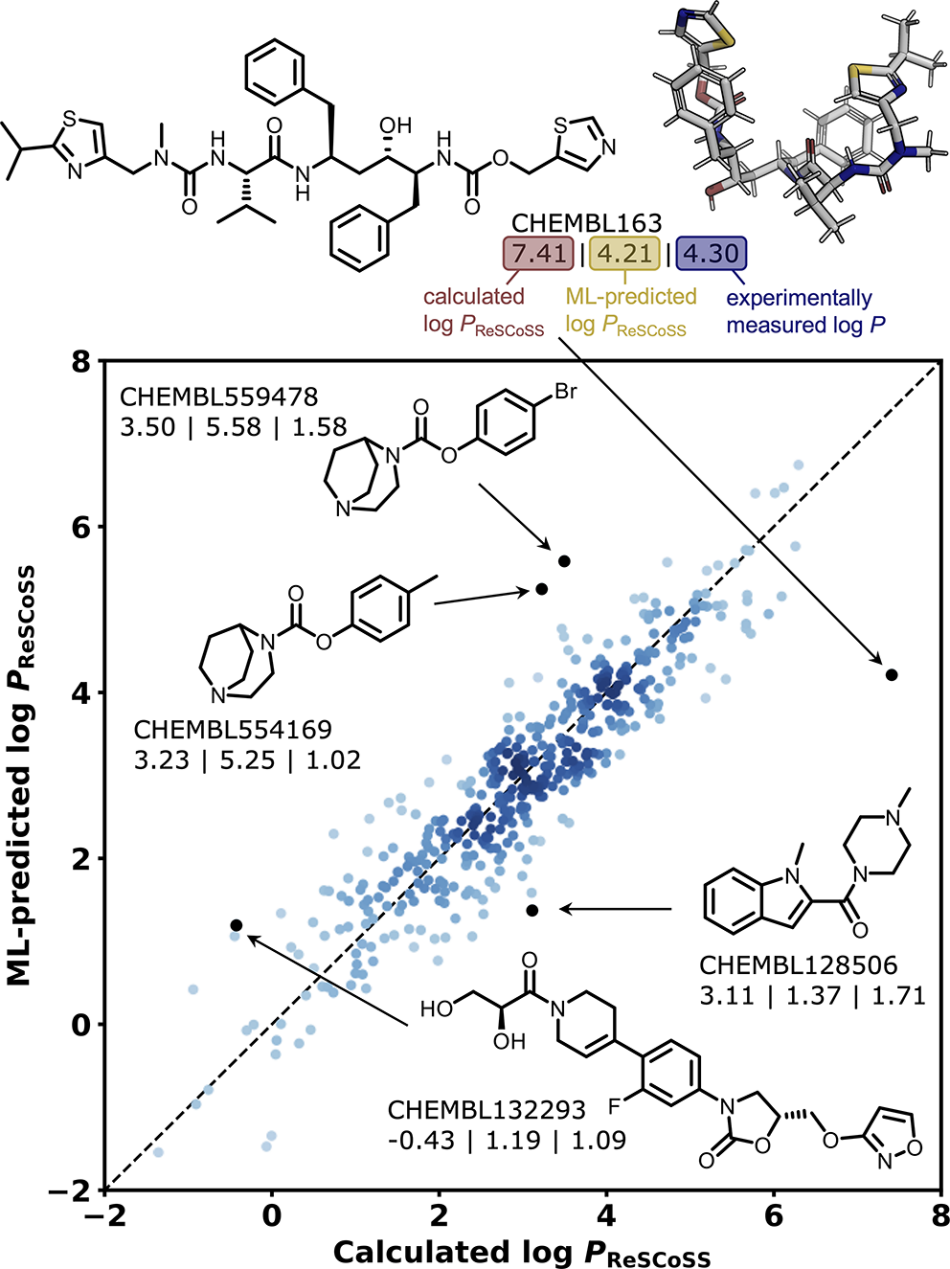
Predicted vs calculated plots for test set molecules from the public data set. Chemprop model trained using a scaffold-based split. Five compounds with the highest absolute deviation from calculated log *P*_ReSCoSS_ values are highlighted. Numbers below each compound specify calculated log *P*_ReSCoSS_ | ML-predicted log *P*_ReSCoSS_ | experimental log *P* (see the top of the figure).

For the best-performing model (Chemprop), we investigate the outliers in this predicted versus calculated plot to gain insights into the failure modes of this model. We place the focus on the scaffold split for the public data set so that full structures can be discussed. Figure 3 shows the five compounds for which the largest absolute deviation between calculated and predicted log *P*_ReSCoSS_ values was observed. These high deviations between predicted and calculated values occur across a range of low, medium, and high calculated lipophilicities. For three of the five selected compounds (CHEMBL554169, CHEMBL559478, and CHEMBL132293), the model overestimates the calculated log *P*_ReSCoSS_ value by up to two log units. The first two compounds constitute a matched molecular pair differing only in the methyl resp. bromo substituent. For these compounds, the ML model substantially overestimates the calculated log *P*_ReSCoSS_ values, and those in turn overestimate the experimentally measured values. Interestingly, however, the relative lipophilicity trend between the two compounds is preserved across the three methods, with both the calculated (0.27 log units) and ML-predicted (0.33 log units) differences approximately matching the experimental one (0.56 log units). For the three remaining compounds (CHEMBL132293, CHEMBL128506, and CHEMBL163), ML-predicted log *P*_ReSCoSS_ values substantially deviate from calculated log *P*_ReSCoSS_ values but approximate experimental measurements better than the calculated values. Particularly for CHEMBL132293 and CHEMBL163, this observation may be connected to the previously observed tendency of the underlying COSMO-RS method to show an increased error for large compounds.^23^ For CHEMBL163 (the approved HIV protease inhibitor Ritonavir^94^), the model predicts a log *P*_ReSCoSS_ of 4.21 (close to the experimental measurement of 4.30), while the calculated value is 7.41. This difference might be caused by the high flexibility of the molecule and the folded shape it adopts in the lowest-energy conformation (DFT, both gas phase and water, Figure 3), shielding polar moieties from the solvent environment and thus expecting greater lipophilicity than the Chemprop model prediction from the 2D graph.

As an initial hypothesis, we suspected that the fact that ML-predicted ReSCoSS values are close to experimental values in the discussed cases may indicate that the model has learned to disregard compounds where the calculated log *P*_ReSCoSS_ values appear as strong outliers. A similar effect was observed for 10 large, flexible compounds from internal discovery projects, for which the ML-predicted log *P*_ReSCoSS_ values matched experimental measurements well [mean absolute error (MAE) = 0.27], while calculated log *P*_ReSCoSS_ values substantially overestimated drug lipophilicity. However, upon closer examination of errors with respect to experimental values for both ML-predicted and calculated log *P*_ReSCoSS_ values (Figure S20), no general trend could be confirmed.

The very similar performances of the Chemprop3D model and the 2D-graph-based Chemprop model implies that the interatomic distances encoded in the 3D graph representation were not helpful for this learning task. An analysis of the hyperparameter screening for the Chemprop3D model (Figure S2 and S3) confirms this hypothesis: The cross-validation error during the hyperparameter screening generally increased with increasing graph cutoff distance. During hyperparameter optimization, the model using a graph cutoff distance of 2 Å emerges as the best-performing model and is tested on the test set, showing very similar performance to the 2D-graph-based model. A 2 Å cutoff distance results in a graph that includes almost exclusively edges, which correspond to covalent bonds. The interatomic distances, which in this case correspond to bond lengths, do not change substantially between the different optimized conformers, and do not appear to add relevant information to the set of bond features in the original Chemprop implementation. This hypothesis is confirmed by the very similar performance when retraining Chemprop3D models using the lowest GFN2-xTB energy in wet octanol or the lowest DFT energy in water as the conformer selection criterion (Figure S4).

### Approximating Experimental log *P* Values

**Figure 4:**
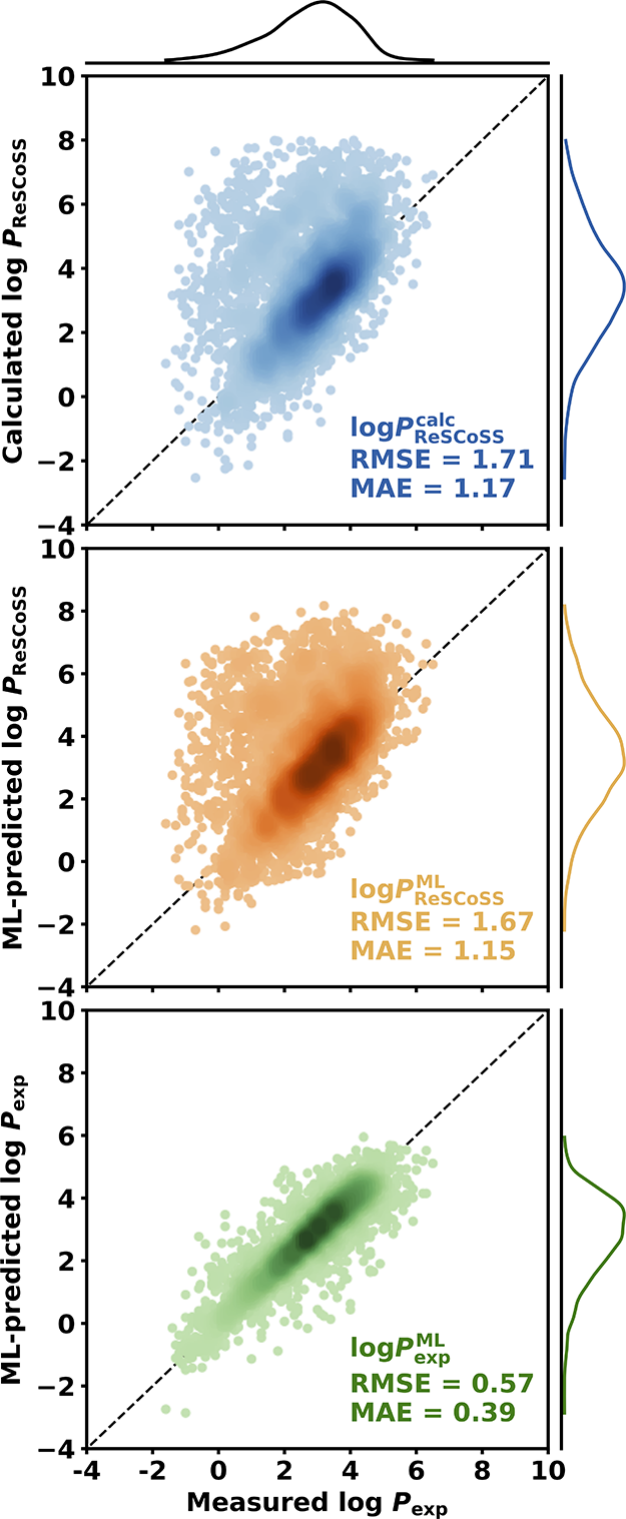
Predicted vs calculated plots for test set molecules from the in-house data set. Chemprop models trained using a scaffold-based split. Calculated log *P*_ReSCoSS_ (top), ML-predicted log *P*_ReSCoSS_ (middle), and ML-predicted log *P*_exp_ (bottom) against experimentally measured log *P*. Kernel density estimates for experimental, ML-predicted, and calculated values shown on the top, respectively, left of panels. Stated errors with respect to experimentally measured log *P*.

Besides investigating the ability of trained models to approximate the quantity that they were trained to predict (log *P*_ReSCoSS_), we additionally investigated the models’ ability to approximate experimentally measured log *P* values (Figures 4 and S10–S14 and Tables S5–S7). The ML models provide a similarly good approximation to experimental values as the ReSCoSS-calculated log *P* values. However, models trained on ReSCoSS-calculated values are limited by the accuracy of the underlying physical method and approximate the experimental values less accurately than ML models directly trained on experimental values.

The theoretical advantages of training models on a physics-based log *P* approximation such as ReSCoSS/COSMO-RS rather than on experimentally determined values are two-fold: first, a log *P* space that cannot be easily measured experimentally [e.g., very lipophilic (log *P* > 4.7) or poorly soluble compounds] can be accessed. Second, the training set can easily be extended to include a larger number of compounds, or compounds from a specific chemical region of interest, for example, at the beginning of a new drug discovery project in a previously underexplored chemical space. However, while additional compounds could be added to the log *P*_ReSCoSS_ training data without the need to synthesize and test them, we stress that the utility of this is based on whether the QM method provides a useful log *P* estimate in the chemical space of interest. Across the overall data sets that were utilized in this work, no advantage of training models on ReSCoSS-calculated log *P* values was observed, though individual areas of chemical space may benefit from such an approach. Experience from internal drug discovery projects shows that the ReSCoSS workflow is often able to either estimate measured log *P* values correctly or at least provide a ranking of new ideas with respect to log *P* values prior to synthesis. This has to be assessed on a project-specific basis, and further studies to investigate general guidelines for individual structure classes are necessary. Assessing the utility of training on synthetic data is further complicated by the fact that existing experimental data for, for example, highly lipophilic compounds might be associated with high experimental errors,^26^ creating a lack of a suitable comparison metric.

As an additional benchmark, we investigated a set of peptides and peptide derivatives with experimental log *D* measurements assembled by Fuchs et al.^95^ By focusing on non-ionizable compounds (as reported in the original work), the reported log *D* measurements may be used as a proxy for log *P* measurements. Excluding charged and zwitterionic compounds leaves 75 peptides for analysis, with a size of 25.4 ± 7.5 heavy atoms (mean ± 1 standard deviation). Testing the performance of our log *P*_ReSCoSS_-trained Chemprop model (scaffold split, trained on in-house data), a root mean square error (RMSE) of 0.77 log units was observed (MAE = 0.65 log units). Due to differences in evaluation criteria (see the Supporting Information for details), a direct comparison between our result and those from Fuchs et al. is difficult. The generally lower error values (RMSE = 0.29–0.75 log units) reported for models trained specifically on peptides^95^ suggest that a purpose-built model may be beneficial for this chemical space. It is noteworthy that our log *P*_ReSCoSS_-trained model achieves a better predictive accuracy on this task than an analogous Chemprop model trained on experimental log *P* values (RMSE = 0.99 and MAE = 0.86), potentially indicating that training on ReSCoSS-calculated values can yield a robust generalization capability (Figure S15 and Table S8).

### Learning Curves

**Figure 5:**
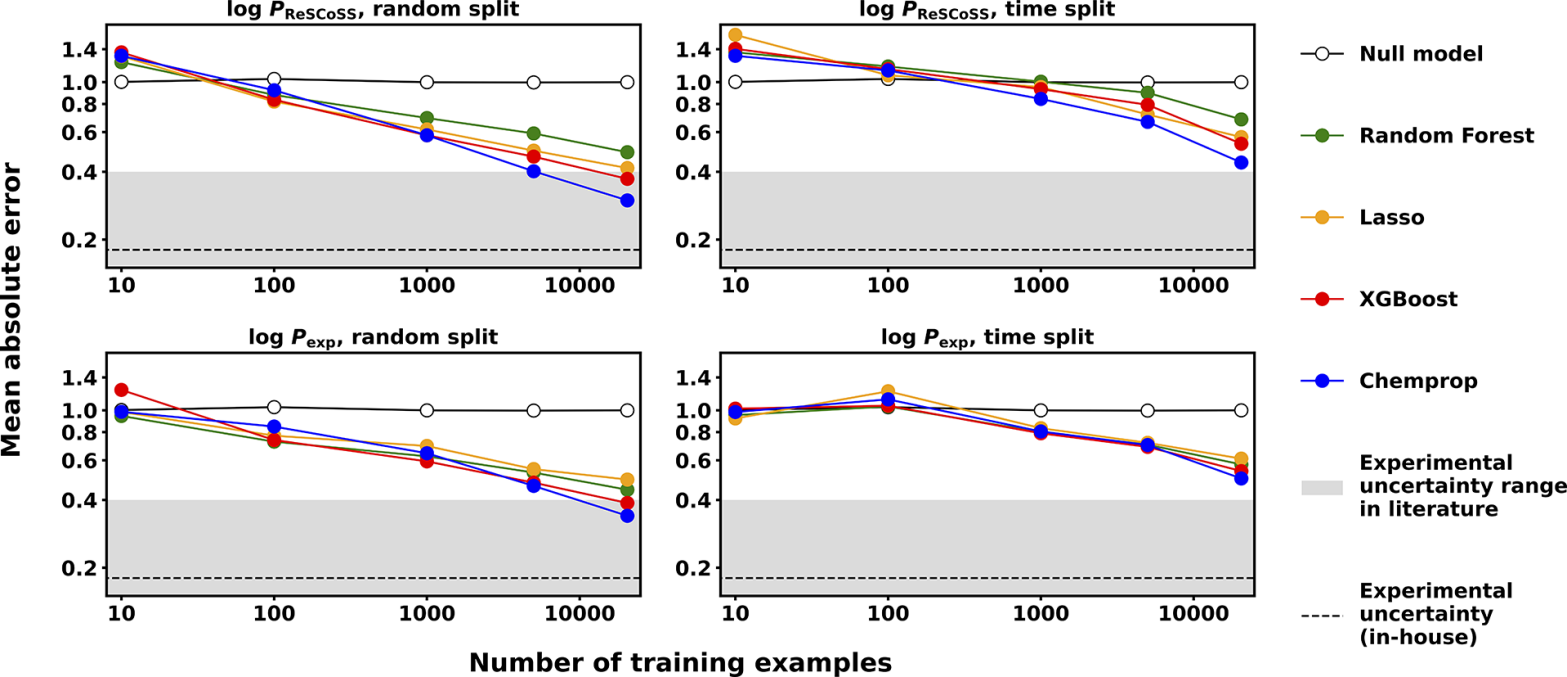
Learning curves for models trained on the in-house data set showing test set MAE vs training set size (10, 100, 1000, 5000, and ∼20,500 compounds). (Top) Prediction of log *P*_ReSCoSS_, random and time splits. (Bottom) Prediction of log *P*_exp_, random and time splits. The null model simply predicts the average of the training set labels for any given number of training examples. Experimental uncertainty range compiled from literature sources is between <0.2 and 0.4 log units.^23,29,30^ Note the logarithmic scale of both axes. All error bars are smaller than the plot markers and have been omitted for visual clarity. See Figure S17 for error bars.

Figure 5 shows learning curves^41^ (MAE on the hold-out test set vs the number of training examples from the in-house data set) for the Random Forest, Lasso, XGBoost, and Chemprop models analyzed in this work. Due to the similar performances of the Chemprop3D models and the Chemprop models (Figure S4) and the associated high training cost, no learning curves were generated for these models. Figure S17 and S18 reveal more details on both in-house and public data set learning curves for different data set splitting strategies. As expected, the test set error generally decreases for all models with increasing training set size. Models trained to predict log *P*_ReSCoSS_ and those trained to predict log *P*_exp_ with a random split show a clear linear decrease in the test set error with training set size, as described by the inverse power law relationship.^77,96,97^ The higher predictive accuracy of Chemprop models over the classical ML models only manifests itself at training set sizes above ∼1000 data points, indicative of the commonly observed behavior of deep learning models requiring a sufficient amount of training data for good modeling performance.^98^ All model architectures outperform the null model (which simply predicts the average of the training set labels for all compounds in the test set) given sufficient training data. This is the case at 100 or fewer training points for random splits and at approximately 1000 training points for the time split.

The log *P*_ReSCoSS_ models trained on a time split (Figure 5, top right) depart from the linear shape of the learning curve as they see a sharp decrease in the test set error when going from 5000 to ∼20,500 training points, indicating that the compounds registered in the months just before the test set compounds provide particularly useful information to the models during the learning process. This finding is in line with the iterative nature of the drug development process. During a discovery project, subsequent compounds are designed based on previous iterations of the same scaffold, or new chemical series might be started that were not present in the smaller (chronologically earlier) training sets.

Only the Chemprop and XGBoost models trained with a random split achieve MAEs on the test set that reach the upper part of the experimental uncertainty range of <0.2–0.4 log units.^23,29,30^ As no clear leveling-off in the learning curves is observed at the largest tested training set sizes, adding additional compounds to the training set is expected to further decrease the test set error. For the log *P*_ReSCoSS_ models, this step merely incurs computational, though no experimental, costs (in contrast to the log *P*_exp_ models). We hypothesize that the test set error for the log *P*_exp_ models will level off once the test set error reaches the level of average experimental uncertainty in these data sets. Compounds for which multiple measurements exist in the in-house data set show an average absolute difference in those measurements of 0.18 log units (Figure 5, see Figure S16 for more details). Accordingly, a larger number of training examples would be required to decrease the test set error to the point that a leveling-off effect due to experimental uncertainty could be observed.

## Conclusions

Different ML models were employed to learn ReSCoSS-calculated log *P* values in order to obtain a computationally cheap, QM-based estimation of drug lipophilicity. Models were benchmarked on both a public data set and an in-house data set. The message-passing neural network model Chemprop yielded the highest predictive accuracy among the tested models with MAEs of 0.44 and 0.34 log units for scaffold splits of the public and in-house data sets, respectively. This model is able to accurately predict ReSCoSS-calculated log *P* values and to approximate experimentally measured log *P* values similarly well as the underlying QM-based method. However, with regard to approximating experimental values, both methods are outperformed by ML models trained directly on experimental values. If reliable experimental values are available, these should be used preferentially to train ML models, while the utility of training on ReSCoSS-calculated log *P* values must be assessed on a project-specific basis.

For both the public and in-house data sets, learning curves indicate that additional training data would likely decrease the test set error further. In contrast to models trained on experimental data, this increase in training set size can be achieved at comparatively low cost by performing additional ReSCoSS/COSMO-RS calculations. Reducing the required computational time from ∼1 h/compound to being able to predict log *P*_ReSCoSS_ values for hundreds of compounds per second (without tautomer selection based on semi-empirical calculations) enables efficient prescreening of large compound collections and prioritization of compounds for full QM calculations. This might accelerate computational analyses for projects that have found ReSCoSS-calculated log *P* values to be useful in their decision-making process.

Though single-conformer geometries did not improve the modeling performance, extensions to this work may focus on utilizing multiconformer representations to more closely mimic the ReSCoSS workflow and on determining the general guidelines for the identification of structure classes for which ReSCoSS-calculated log *P* values constitute suitable synthetic training data.

## Supporting Information Available

Detailed plots on results as described in the manuscript (PDF)
