# Supplementary material for: Machine Learning for Fast, Quantum Mechanics-Based Approximation of Drug Lipophilicity
Source: ACS Omega. 2023 Jan 4;8(2):2046–56. doi: 10.1021/acsomega.2c05607 (PMC9850743; doi:10.1021/acsomega.2c05607)
Supplement: Supplementary file 1 — ao2c05607_si_001.pdf [file ao2c05607_si_001.pdf]

Supporting Information for "Machine learning for fast, quantum mechanics-based approximation of drug lipophilicity"

Clemens Isert [a,b], *Jimmy C. Kromann* [b], *Nikolaus Stiefl* [b], *Gisbert Schneider* [a,c], *Richard A. Lewis* [b,*]

[a] ETH Zurich, Department of Chemistry and Applied Biosciences, Vladimir-Prelog-Weg 4, 8093 Zurich, Switzerland

[b] Novartis Institutes for BioMedical Research, CH-4002 Basel, Switzerland

[c] ETH Singapore SEC Ltd., 1 CREATE Way, #06-01 CREATE Tower, Singapore 138602, Singapore

* Email: richard.lewis@novartis.com

Contents

1) Distribution of measured log *P* values S2
2) Dataset splitting S2
3) Hyperparameter optimization S3
4) Impact of conformer choice on Chemprop3D model performance S5
5) Overall model performance S6
6) Predicted-vs-calculated plots for all models S8
7) Approximating experimental log P values S9
8) log *P* prediction for peptides S11
9) Experimental uncertainty for in-house dataset S12
10) Learning curves S13
11) Flowchart of ReSCoSS workflow S14
12) Errors of ML-predicted and calculated log $P_{ReSCoSS}$ values w.r.t experimental values S14
13) Tanimoto similarities in public & in-house datasets S15
14) Beyond-Rule-of-5 compounds in the public & in-house datasets S15
15) Duplicates between datasets & label consistency S16

1) Distribution of measured log *P* values

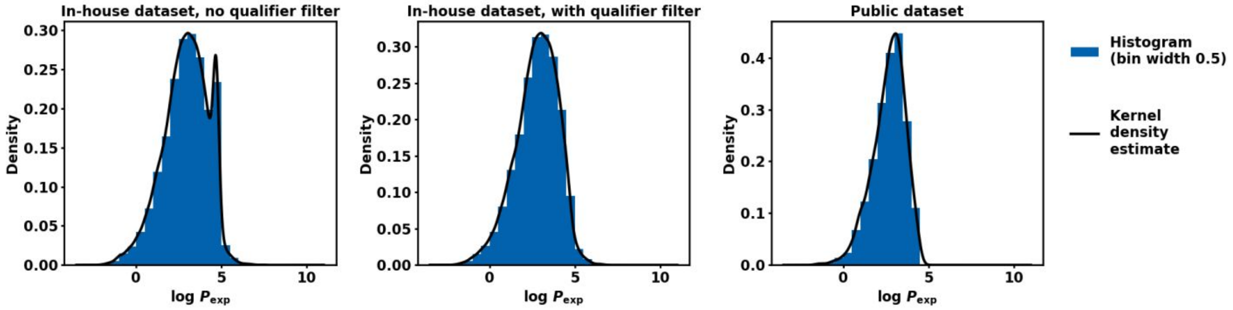

Figure S1 Distribution of measured experimental values for the in-house and public dataset. When no filters on qualifiers (“<”/”>”) are used (left plot), the peak at 4.7 is clearly visible. Applying this filter (middle plot) removes this artefact of the dataset. Approximately 12% of compounds were filtered out due to qualifiers on the measurement.

2) Dataset splitting

For the random splitting, molecules were chosen randomly and assigned to the respective dataset splits. The scaffold-based split was obtained by grouping molecules by their Bemis-Murcko scaffolds[1] based on code from Chemprop[2,3]. Statistics for the individual subsets of the public dataset are shown in Table S1. Clusters of molecules sharing the same Bemis-Murcko scaffold were randomly assigned to the respective dataset splits. For the time-based split, the newest (as determined by internal registration date) 20% of molecules were assigned to the hold-out test set. A five-fold cross-validation scheme (rolling basis) on time series data[4] was used for the hyperparameter optimization, training on increasingly longer time periods while always validating on the subsequent (later registered) 20% of the cross-validation set. In this way, models are only trained on previous data and used to predict later data.

Table S1 Scaffold statistics for public dataset.

Data subset	Number of molecules	Number of scaffolds	Mean number of molecules per scaffold
Test set	424	280	1.51
Fold 0 validation set	338	197	1.72
Fold 1 validation set	338	208	1.63
Fold 2 validation set	338	228	1.48
Fold 3 validation set	338	185	1.83
Fold 4 validation set	338	200	1.69

3) Hyperparameter optimization

For each model, key hyperparameters were optimized using a five-fold cross-validation scheme and a grid-based search strategy. The following hyperparameters were investigated:

- Random Forest
  - Number of estimators: [10, 20, 30, 40, 50, 60, 70, 80, 90, 100, 200, 300, 400, 500, 1000, 2000, 3000]
- Lasso regression
  - Alpha: [0.00001, 0.0001, 0.001, 0.01, 0.1, 1.0]
- XGBoost
  - Maximum depth: [3, 6, 10, 20]
  - Learning rate: [0.01, 0.05, 0.1, 0.3]
  - Number of estimators: [100, 500, 1000]
- Chemprop
  - Number of message passing steps: [2, 3, 4]
  - Hidden size of message passing layers: [300, 800, 1200]
- Chemprop3D
  - Number of message passing steps: [2, 3, 4]
  - Hidden size of message passing layers: [300, 800, 1200]
  - Cutoff distance for 3D graph construction (Å): [2, 4, 6, 9999]
    (9999 results in a fully-connected graph with all interatomic distances)

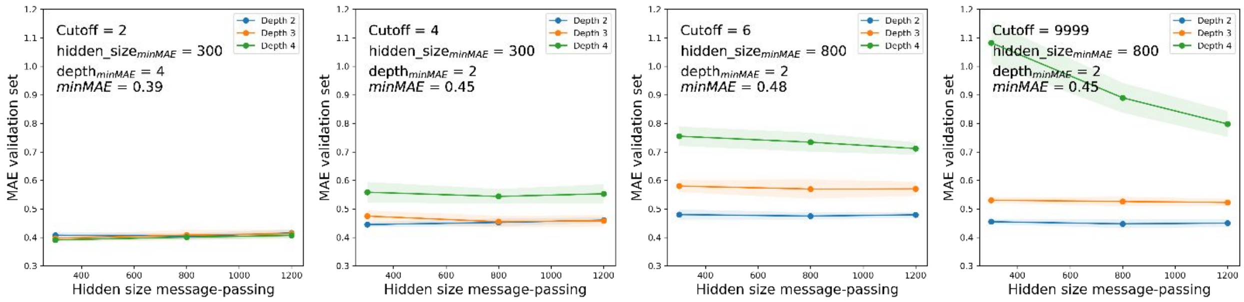

Figure S2 Hyperparameter optimization results for Chemprop3D model (conformer choice based on lowest GFN2-xTB energy in water). Public dataset, random split. Panels show mean absolute error on the validation set during five-fold cross validation, plotted against the hidden size for message passing layers. Cutoff distance for the 3D graph construction is held constant for each panel and increases between panels from left to right. Shaded regions show standard deviation of the error across five folds.

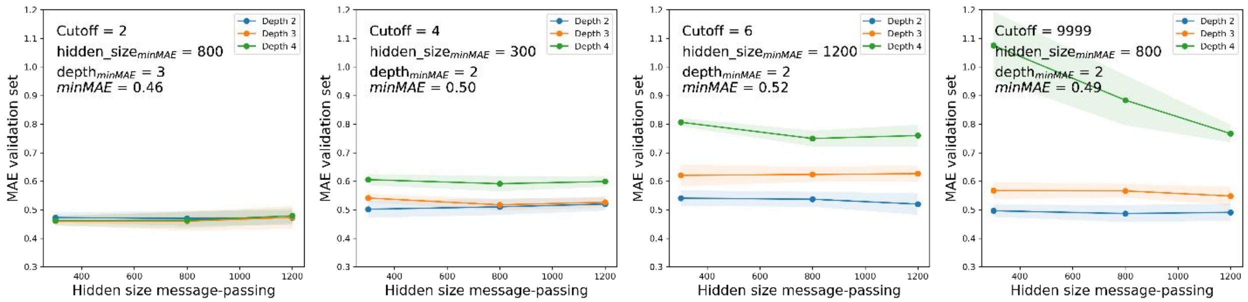

Figure S3 Hyperparameter optimization results for Chemprop3D model (conformer choice based on lowest GFN2-xTB energy in water). Public dataset, scaffold split. Panels show mean absolute error on the validation set during five-fold cross validation, plotted against the hidden size for message passing layers. Cutoff distance for the 3D graph construction is held constant for each panel and increases between panels from left to right. Shaded regions show standard deviation of the error across five folds.

4) Impact of conformer choice on Chemprop3D model performance

Since the cutoff distance for the 3D graph construction was used as an optimization hyperparameter for the Chemprop3D model, it gives some insight into whether this architecture can benefit from 3D information for this learning task. Chemprop3D models were also trained using the lowest GFN2-xTB[5] energy in wet octanol or the lowest DFT energy in water as the conformer selection criterion. For these models, the best-performing hyperparameter setup for models trained on conformers with the lowest GFN2-xTB energy in water was utilized.

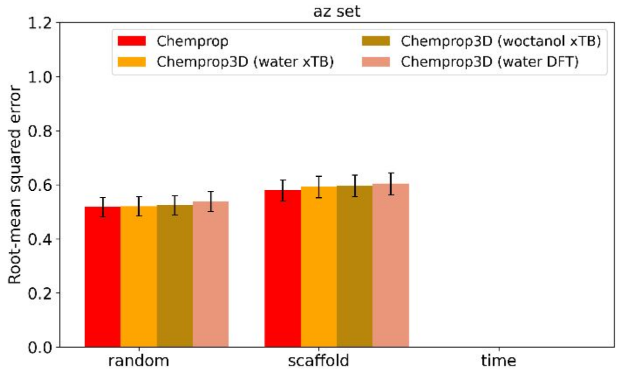

Figure S4 Test set errors for the public dataset with different splitting strategies. Error bars show 95% confidence intervals.[6] All models are using hyperparameters obtained from the cross-validation hyperparameter screening using lowest GFN2-xTB energy in solvent as the criterion for conformer choice.

5) Overall model performance

Table S2 Test set errors (MAE) for the in-house and public datasets with different splitting strategies. Models trained on log $P_{ReSCoSS}$. M: Mean error value. LE: lower error on MAE for 95% confidence interval. UE: upper error on MAE for 95% confidence interval.[6] Chemprop3D trained on conformer with lowest GFN2-xTB energy in water.

Method	In-house dataset									Public dataset					
	Random split			Scaffold split			Time split			Random split			Scaffold split		
	M	LE	UE	M	LE	UE	M	LE	UE	M	LE	UE	M	LE	UE
RF	0.49	0.01	0.01	0.54	0.01	0.01	0.68	0.01	0.01	0.54	0.04	0.04	0.55	0.04	0.04
Lasso	0.42	0.01	0.01	0.44	0.01	0.01	0.57	0.01	0.01	0.51	0.04	0.03	0.50	0.03	0.03
XGBoost	0.37	0.01	0.01	0.40	0.01	0.01	0.53	0.01	0.01	0.46	0.03	0.03	0.46	0.03	0.03
Chemprop	0.30	0.01	0.01	0.34	0.01	0.01	0.44	0.01	0.01	0.38	0.03	0.02	0.44	0.03	0.03
Chemprop3D	-	-	-	-	-	-	-	-	-	0.39	0.03	0.03	0.45	0.03	0.03

Table S3 Test set errors (RMSE) for the in-house and public datasets with different splitting strategies. Models trained on log $P_{ReSCoSS}$. M: Mean error value. LE: lower error on RMSE for 95% confidence interval. UE: upper error on RMSE for 95% confidence interval.[6] Chemprop3D trained on conformer with lowest GFN2-xTB energy in water.

Method	In-house dataset									Public dataset					
	Random split			Scaffold split			Time split			Random split			Scaffold split		
	M	LE	UE	M	LE	UE	M	LE	UE	M	LE	UE	M	LE	UE
RF	0.65	0.01	0.01	0.70	0.01	0.01	0.86	0.02	0.02	0.75	0.05	0.05	0.71	0.05	0.05
Lasso	0.54	0.01	0.01	0.58	0.01	0.01	0.72	0.01	0.01	0.68	0.05	0.04	0.66	0.05	0.04
XGBoost	0.50	0.01	0.01	0.54	0.01	0.01	0.68	0.01	0.01	0.63	0.04	0.04	0.61	0.04	0.04
Chemprop	0.42	0.01	0.01	0.46	0.01	0.01	0.58	0.01	0.01	0.52	0.04	0.03	0.58	0.04	0.04
Chemprop3D	-	-	-	-	-	-	-	-	-	0.52	0.04	0.03	0.59	0.04	0.04

Table S4 Rank-order correlation coefficients (Spearman's ρ and Kendall's τ) for the in-house and public datasets with different splitting strategies. Models trained on log $P_{ReSCoSS}$. Chemprop3D trained on conformer with lowest GFN2-xTB energy in water.

Method	In-house dataset						Public dataset			
	Random split		Scaffold split		Time split		Random split		Scaffold split	
	ρ	τ	ρ	τ	ρ	τ	ρ	τ	ρ	τ
RF	0.9161	0.7556	0.9020	0.7327	0.8860	0.7045	0.8172	0.6341	0.8616	0.6825
Lasso	0.9403	0.7932	0.9298	0.7774	0.9232	0.7572	0.8550	0.6754	0.8888	0.7177
XGBoost	0.9502	0.8138	0.9423	0.7993	0.9273	0.7676	0.8659	0.6939	0.9039	0.7362
Chemprop	0.9652	0.8495	0.9593	0.8344	0.9494	0.8086	0.9147	0.7590	0.9217	0.7587
Chemprop3D	-	-	-	-	-	-	0.9101	0.7477	0.9134	0.7448

Table S5 Test set errors (MAE) for the in-house and public datasets with different splitting strategies. Models trained on log P_{exp}. M: Mean error value. LE: lower error on MAE for 95% confidence interval. UE: upper error on MAE for 95% confidence interval.[6] No Chemprop3D models were trained for this task.

Method	In-house dataset									Public dataset					
	Random split			Scaffold split			Time split			Random split			Scaffold split		
	M	LE	UE	M	LE	UE	M	LE	UE	M	LE	UE	M	LE	UE
RF	0.44	0.01	0.01	0.51	0.01	0.01	0.57	0.01	0.01	0.45	0.03	0.03	0.43	0.03	0.03
Lasso	0.49	0.01	0.01	0.52	0.01	0.01	0.61	0.01	0.01	0.44	0.03	0.03	0.46	0.03	0.03
XGBoost	0.39	0.01	0.01	0.44	0.01	0.01	0.54	0.01	0.01	0.41	0.03	0.03	0.40	0.03	0.03
Chemprop	0.34	0.01	0.01	0.39	0.01	0.01	0.50	0.01	0.01	0.38	0.03	0.02	0.38	0.03	0.02

Table S6 Test set errors (RMSE) for the in-house and public datasets with different splitting strategies. Models trained on log P_{exp}. M: Mean error value. LE: lower error on RMSE for 95% confidence interval. UE: upper error on RMSE for 95% confidence interval.[6] No Chemprop3D models were trained for this task.

Method	In-house dataset									Public dataset					
	Random split			Scaffold split			Time split			Random split			Scaffold split		
	M	LE	UE	M	LE	UE	M	LE	UE	M	LE	UE	M	LE	UE
RF	0.63	0.01	0.01	0.71	0.01	0.01	0.77	0.01	0.01	0.58	0.04	0.04	0.54	0.04	0.04
Lasso	0.68	0.01	0.01	0.72	0.01	0.01	0.81	0.02	0.02	0.63	0.04	0.04	0.60	0.04	0.04
XGBoost	0.56	0.01	0.01	0.64	0.01	0.01	0.73	0.01	0.01	0.55	0.04	0.04	0.52	0.04	0.03
Chemprop	0.51	0.01	0.01	0.57	0.01	0.01	0.69	0.01	0.01	0.52	0.04	0.03	0.52	0.04	0.03

Table S7 Rank-order correlation coefficients (Spearman's ρ and Kendall's τ) for the in-house and public datasets with different splitting strategies. Models trained on log P_{exp}. No Chemprop3D models were trained for this task.

Method	In-house dataset						Public dataset			
	Random split		Scaffold split		Time split		Random split		Scaffold split	
	ρ	τ	ρ	τ	ρ	τ	ρ	τ	ρ	τ
RF	0.8642	0.6986	0.8210	0.6521	0.7726	0.5911	0.7886	0.5979	0.7971	0.6074
Lasso	0.8470	0.6732	0.8192	0.6464	0.7550	0.5731	0.7810	0.6062	0.7900	0.5974
XGBoost	0.8962	0.7422	0.8604	0.6990	0.8006	0.6219	0.8117	0.6290	0.8064	0.6175
Chemprop	0.9131	0.7719	0.8881	0.7363	0.8262	0.6535	0.8323	0.6575	0.8159	0.6416

6) Predicted-vs-calculated plots for all models

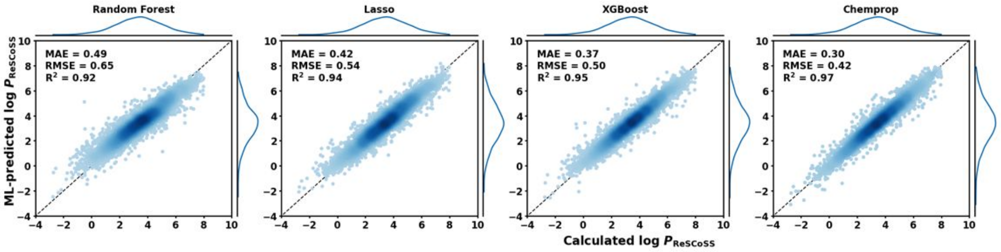

Figure S5 Predicted-vs-calculated plots for test set molecules. In-house dataset, random split.

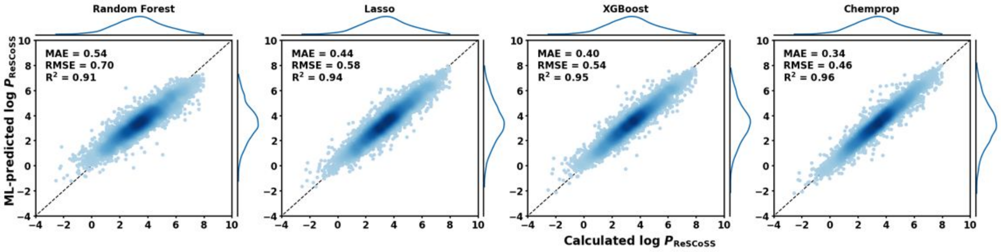

Figure S6 Predicted-vs-calculated plots for test set molecules. In-house dataset, scaffold split.

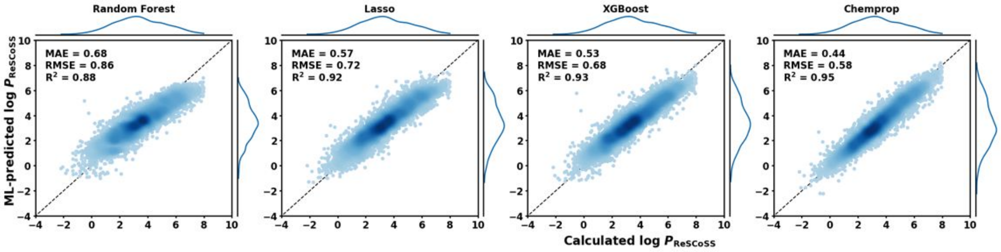

Figure S7 Predicted-vs-calculated plots for test set molecules. In-house dataset, time split.

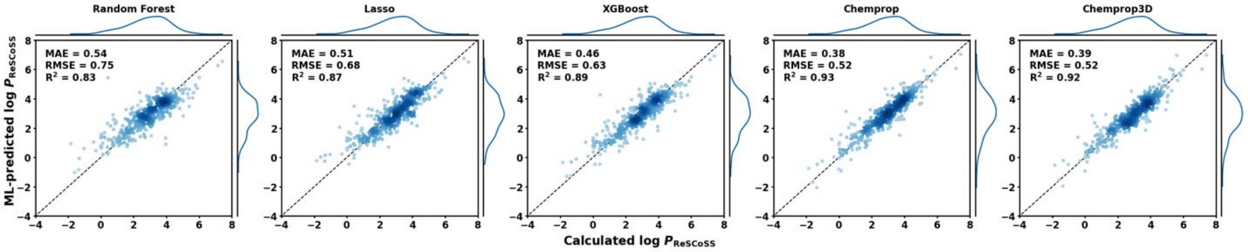

Figure S8 Predicted-vs-calculated plots for test set molecules. Public dataset, random split.

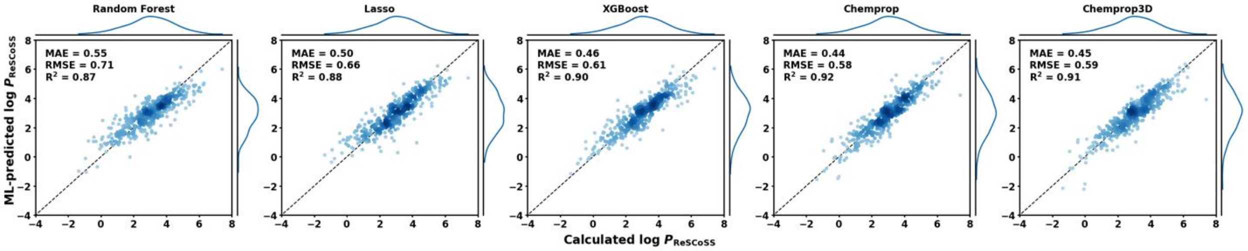

Figure S9 Predicted-vs-calculated plots for test set molecules. Public dataset, scaffold split.

7) Approximating experimental log P values

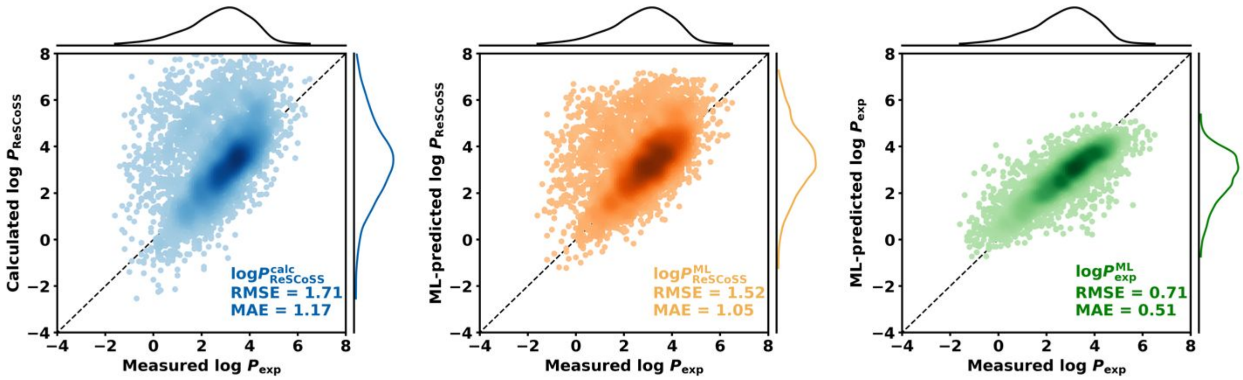

Figure S10 Predicted-vs-calculated plots for test set molecules from the in-house dataset. Random Forest models trained using scaffold-based split. (Left): Calculated log $P_{ReSCoSS}$ (blue) and ML-predicted log $P_{ReSCoSS}$ (orange) against experimentally measured log *P*. (Right): ML-predicted log *P* against experimentally measured log *P*. Kernel-density estimates for experimental, ML-predicted, and calculated values shown on the left and top of each panel, respectively.

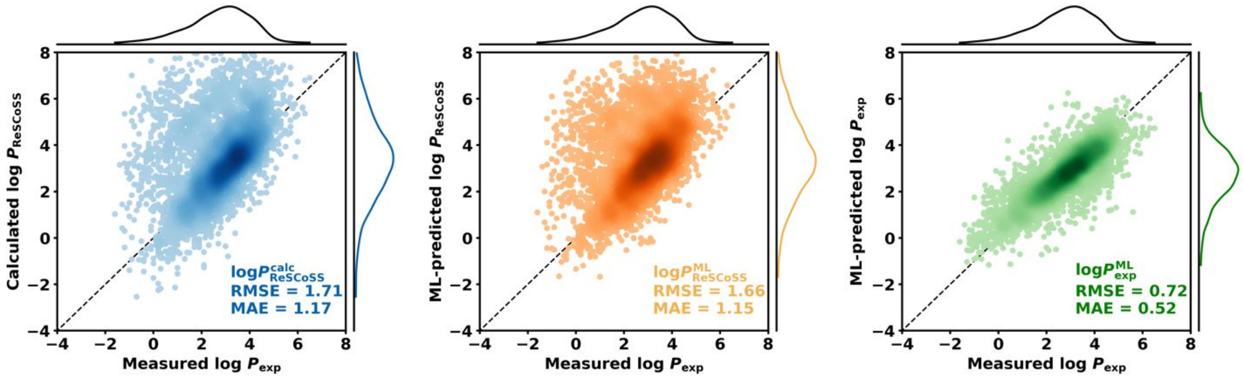

Figure S11 Predicted-vs-calculated plots for test set molecules from the in-house dataset. Lasso models trained using scaffold-based split. (Left): Calculated log $P_{ReSCoSS}$ (blue) and ML-predicted log $P_{ReSCoSS}$ (orange) against experimentally measured log *P*. (Right): ML-predicted log *P* against experimentally measured log *P*. Kernel-density estimates for experimental, ML-predicted, and calculated values shown on the left and top of each panel, respectively.

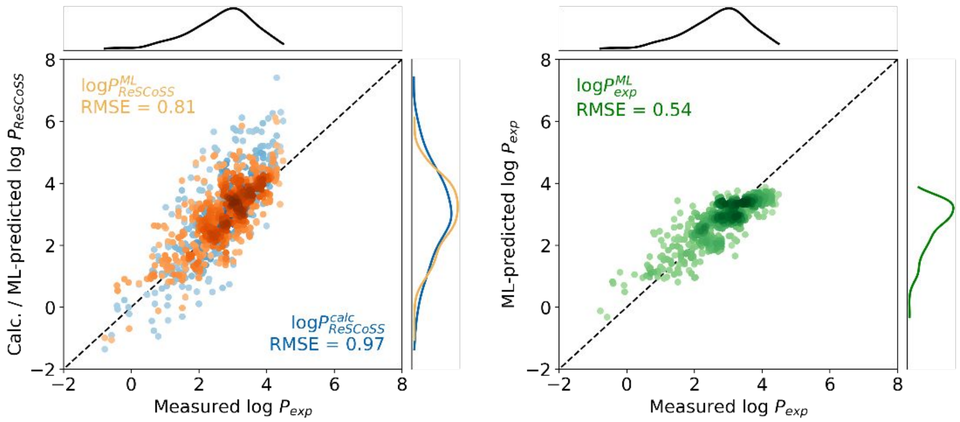

Figure S12 Predicted-vs-calculated plots for test set molecules from the public dataset. Random Forest models trained using scaffold-based split. (Left): Calculated log P_{ReSCoSS} (blue) and ML-predicted log P_{ReSCoSS} (orange) against experimentally measured log *P*. (Right): ML-predicted log *P* against experimentally measured log *P*. Kernel-density estimates for experimental, ML-predicted, and calculated values shown on the left and top of each panel, respectively.

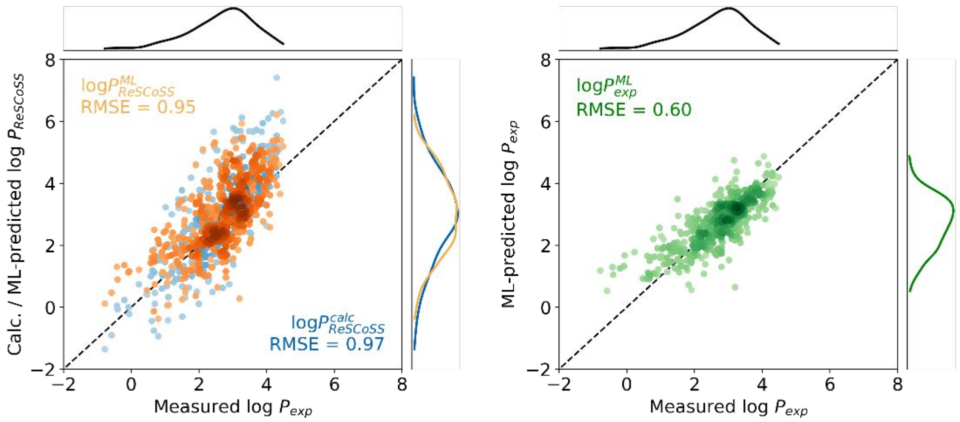

Figure S13 Predicted-vs-calculated plots for test set molecules from the public dataset. Lasso models trained using scaffold-based split. (Left): Calculated log P_{ReSCoSS} (blue) and ML-predicted log P_{ReSCoSS} (orange) against experimentally measured log *P*. (Right): ML-predicted log *P* against experimentally measured log *P*. Kernel-density estimates for experimental, ML-predicted, and calculated values shown on the left and top of each panel, respectively.

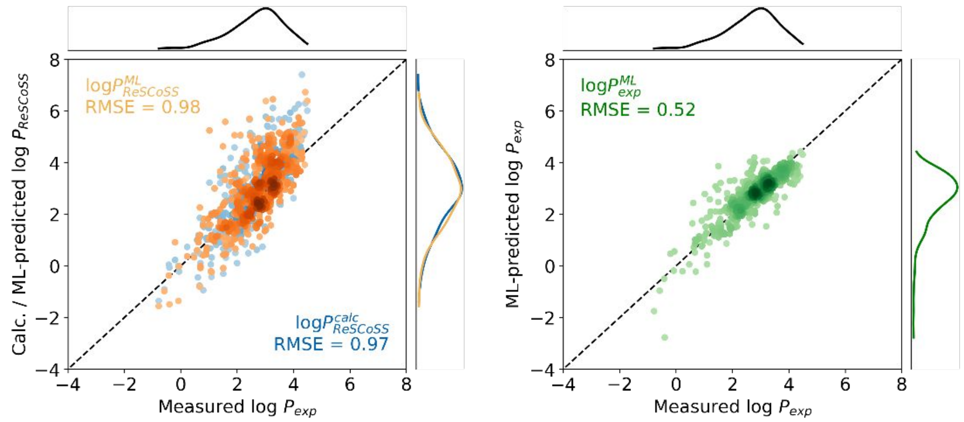

Figure S14 Predicted-vs-calculated plots for test set molecules from the public dataset. Chemprop models trained using scaffold-based split. (Left): Calculated log P_{ReSCoSS} (blue) and ML-predicted log P_{ReSCoSS} (orange) against experimentally measured log *P*. (Right): ML-predicted log *P* against experimentally measured log *P*. Kernel-density estimates for experimental, ML-predicted, and calculated values shown on the left and top of each panel, respectively.

8) log *P* prediction for peptides

SMILES for the set of LIPOPEP compounds were extracted from the ESI of the reference publication.[7] While the publication reports RMSE separately for the cross-validation and test set ("external validation"), this split is not provided in the ESI and we test our models (trained on the in-house data) on the entire LIPOPEP dataset after filtering steps. Note that no re-training of our models was performed. We exclude compounds which the authors denote as "ionizable" and further remove charged or zwitterionic compounds as described in section "Methods". No further SMILES preprocessing (tautomer selection etc.) was performed for this analysis. Model predictions are made using the Chemprop[2,3] model trained on in-house data using a scaffold-based split. Table S6 and Figure S15 show prediction results.

Table S8 Test set errors for the peptide dataset (LIPOPEP)[7]. Errors reported w.r.t experimental measurements. Models trained on in-house data using scaffold split. M: Mean error value. LE: lower error on error metric for 95% confidence interval. UE: upper error on error metric for 95% confidence interval.[6]

	MAE			RMSE		
	M	LE	UE	M	LE	UE
Chemprop (log P_{ReSCoSS})	0.65	0.11	0.10	0.77	0.14	0.12
Chemprop (log P_{exp})	0.86	0.15	0.13	0.99	0.17	0.15

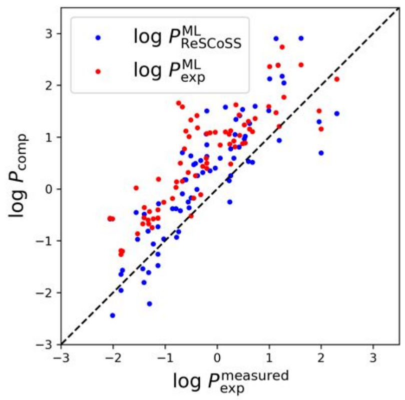

Figure S15 Predicted-vs-calculated plots for peptide molecules. Chemprop models trained on in-house data using scaffold-based split.

9) Experimental uncertainty for in-house dataset

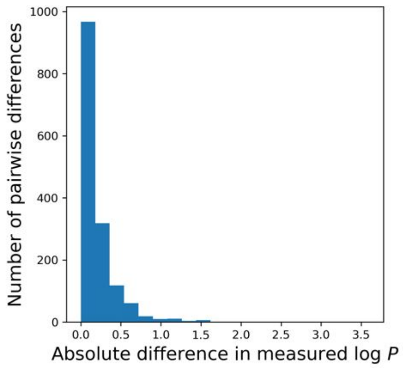

Figure S16 Histogram of absolute differences between pairs of compounds with multiple experimental log *P* measurements for in-house dataset. In case of >2 measurements, all pairwise combinations are considered (1518 total). Average deviation between all pairwise differences is 0.18 log units.

10) Learning curves

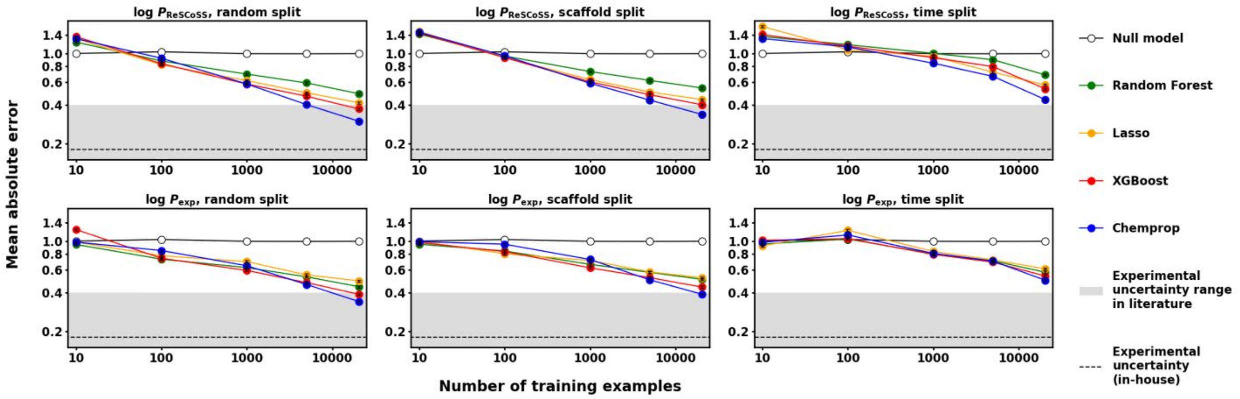

Figure S17 Learning curves for models trained on the in-house dataset showing test set mean absolute error vs. training set size (10, 100, 1000, 5000, and ~20500 compounds). (Top) Prediction of log P_{ReSCoSS}, random, scaffold and time split. (Bottom) Prediction of log P_{exp}, random, scaffold and time split. The null model simply predicts the average of the training set labels for any given number of training examples. Experimental uncertainty range compiled from literature sources is between <0.2 and 0.4 log units.[8–10] Note the logarithmic scale of both axes. Error bars show 95% confidence interval.[6]

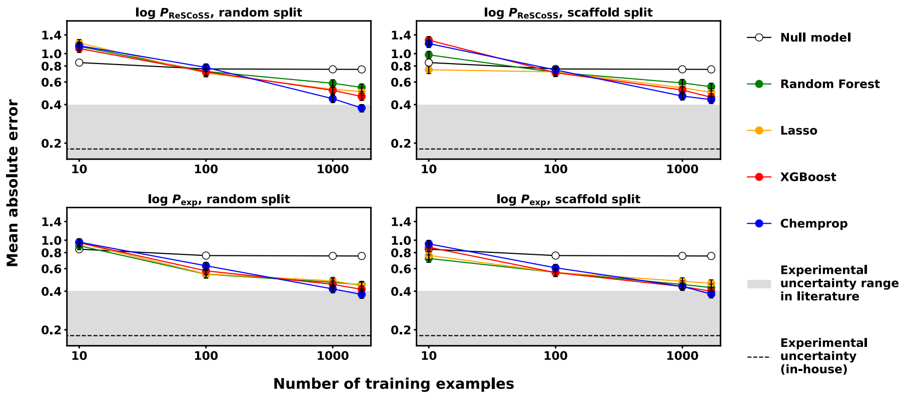

Figure S18 Learning curves for models trained on the public dataset showing test set mean absolute error vs. training set size (10, 100, 1000, and ~1700 compounds). (Top) Prediction of log P_{ReSCoSS}, random and scaffold split. (Bottom) Prediction of log P_{exp}, random and scaffold split. The null model simply predicts the average of the training set labels for any given number of training examples. Experimental uncertainty range compiled from literature sources is between <0.2 and 0.4 log units.[8–10] Note the logarithmic scale of both axes. Error bars show 95% confidence interval.[6]

11) Flowchart of ReSCoSS workflow

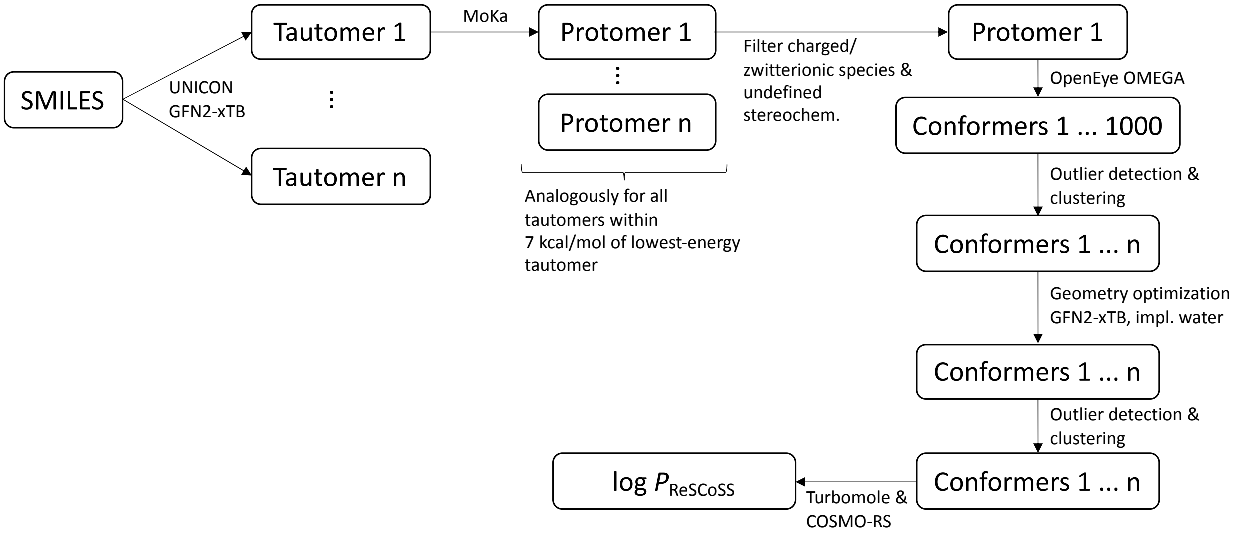

Figure S19 Flowchart of the ReSCoSS workflow. See Methods section for a detailed description.

12) Errors of ML-predicted and calculated log $P_{ReSCoSS}$ values w.r.t experimental values

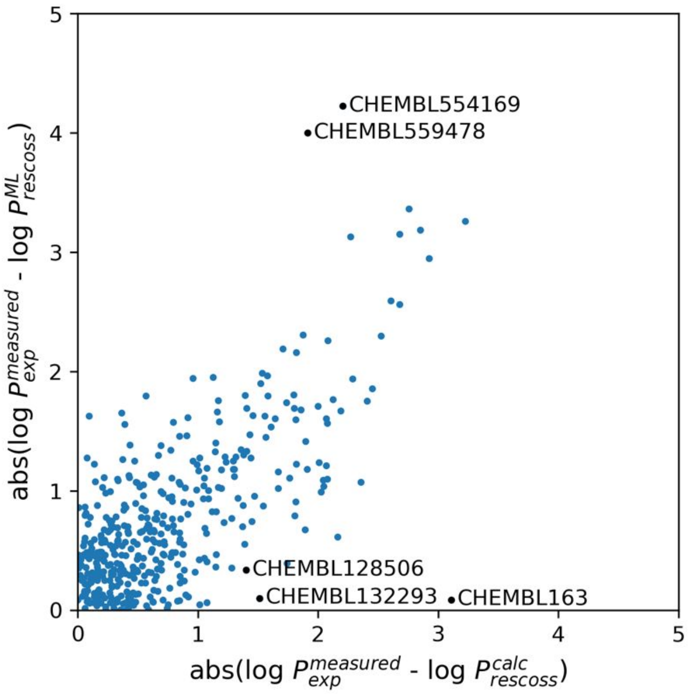

Figure S20 Absolute errors of ML-predicted log $P_{ReSCoSS}$ values vs absolute errors of calculated log $P_{ReSCoSS}$ values, both w.r.t experimental values. The five compounds highlighted in Figure 3 in the main manuscript are highlighted here as well. Test set molecules of the scaffold split for the public dataset shown. ML-predicted log $P_{ReSCoSS}$ values are obtained using the trained Chemprop model.

13) Tanimoto similarities in public & in-house datasets

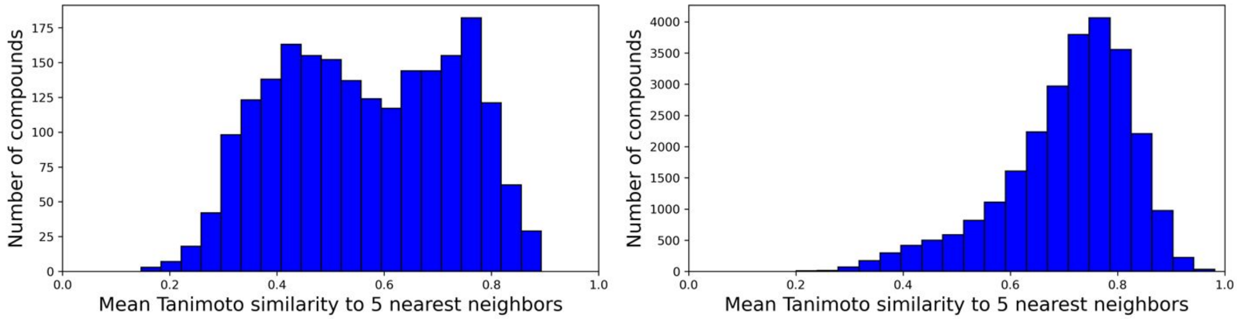

Figure S21 Distribution of mean Tanimoto similarity (radius 2 Morgan Fingerprints, count-based, 2048-bit) between compounds of the public (left) and in-house (right) datasets and their 5 nearest neighbors within the respective datasets. Public dataset shows mean similarity of 0.5666 ± 0.1636, in-house dataset 0.7069 ± 0.1196 (mean ± 1 std. deviation).

14) Beyond-Rule-of-5 compounds in the public & in-house datasets

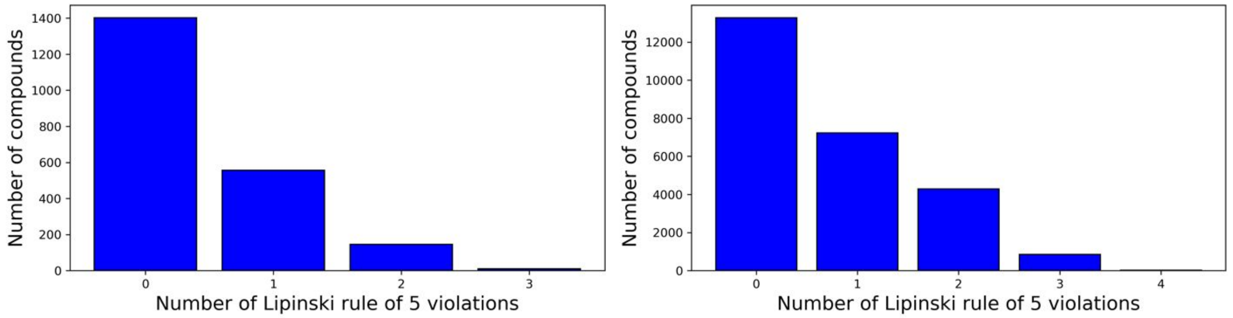

Figure S22 Distribution of the number of violations of Lipinski's Rule-of-5 for the public dataset (left) and inhouse dataset (right).[11]

15) Duplicates between datasets & label consistency

There are 29 duplicate compounds (same canonical SMILES) between the public and inhouse datasets which were analyzed for the consistency in their labels, both for experimental and ReSCoSS-calculated log *P* values (Figure S23). For experimentally determined values, most compounds show only small deviations between both datasets. We attribute these to experimental uncertainty and differences in the measurement protocol and equipment used in the collection of both datasets. The few larger deviations could be caused by experimental errors. In the case of ReSCoSS-calculated values, differences in the labels are at the level of numerical accuracy (mean difference 0.0022).

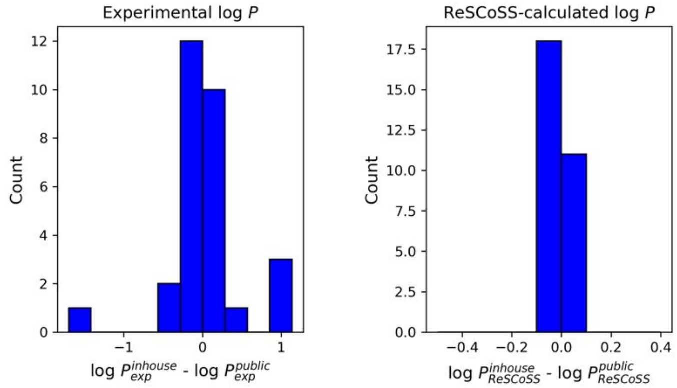

Figure S23 Distribution of difference in labels between the inhouse and public dataset, for experimental (left) and ReSCoSS-calculated (right) log *P* values.

References

(1) Bemis, G. W.; Murcko, M. A. The Properties of Known Drugs. 1. Molecular Frameworks. *J. Med. Chem.* **1996**, *39* (15), 2887–2893. https://doi.org/10.1021/jm9602928.

(2) Yang, K.; Swanson, K.; Jin, W.; Coley, C.; Eiden, P.; Gao, H.; Guzman-Perez, A.; Hopper, T.; Kelley, B.; Mathea, M.; Palmer, A.; Settels, V.; Jaakkola, T.; Jensen, K.; Barzilay, R. Analyzing Learned Molecular Representations for Property Prediction. *J. Chem. Inf. Model.* **2019**, *59* (8), 3370–3388. https://doi.org/10.1021/acs.jcim.9b00237.

(3) Jin, W.; Swanson, K.; Yang, K.; Barzilay, R.; Jaakkola, T. *Chemprop*. https://github.com/chemprop/chemprop (accessed 2022-06-08).

(4) Shrivastava, S. *Cross Validation in Time Series*. https://medium.com/@soumyachess1496/cross-validation-in-time-series-566ae4981ce4 (accessed 2022-04-25).

(5) Bannwarth, C.; Ehlert, S.; Grimme, S. GFN2-XTB - An Accurate and Broadly Parametrized Self-Consistent Tight-Binding Quantum Chemical Method with Multipole Electrostatics and Density-Dependent Dispersion Contributions. *J. Chem. Theory Comput.* **2019**, *15* (3), 1652–1671. https://doi.org/10.1021/acs.jctc.8b01176.

(6) Jensen, J. H. *Which Method Is More Accurate? Or Errors Have Error Bars*; preprint; PeerJ Preprints, 2017. https://doi.org/10.7287/peerj.preprints.2693v1.

(7) Fuchs, J.-A.; Grisoni, F.; Kossenjans, M.; Hiss, J. A.; Schneider, G. Lipophilicity Prediction of Peptides and Peptide Derivatives by Consensus Machine Learning. *MedChemComm* **2018**, *9* (9), 1538–1546. https://doi.org/10.1039/C8MD00370J.

(8) Ulrich, N.; Goss, K. U.; Ebert, A. Exploring the Octanol–Water Partition Coefficient Dataset Using Deep Learning Techniques and Data Augmentation. *Commun. Chem.* **2021**, *4* (1), 1–10. https://doi.org/10.1038/s42004-021-00528-9.

(9) Aliagas, I.; Gobbi, A.; Lee, M.-L.; Sellers, B. D. Comparison of LogP and LogD Correction Models Trained with Public and Proprietary Data Sets. *J. Comput. Aided Mol. Des.* **2022**, *36* (3), 253–262. https://doi.org/10.1007/s10822-022-00450-9.

(10) Bannan, C. C.; Calabró, G.; Kyu, D. Y.; Mobley, D. L. Calculating Partition Coefficients of Small Molecules in Octanol/Water and Cyclohexane/Water. *J. Chem. Theory Comput.* **2016**, *12* (8), 4015–4024. https://doi.org/10.1021/acs.jctc.6b00449.

(11) Lipinski, C. A.; Lombardo, F.; Dominy, B. W.; Feeney, P. J. Experimental and Computational Approaches to Estimate Solubility and Permeability in Drug Discovery and Development Settings. *Adv. Drug Deliv. Rev.* **2012**, *64*, 4–17. https://doi.org/10.1016/j.addr.2012.09.019.
